# Foundation Level Barriers to the Widespread Adoption of Digital Solutions by Care Homes: Insights from Three Scottish Studies

**DOI:** 10.3390/ijerph19127407

**Published:** 2022-06-16

**Authors:** Lucy Johnston, Heidi Koikkalainen, Lynda Anderson, Paul Lapok, Alistair Lawson, Susan D. Shenkin

**Affiliations:** 1 School of Health & Social Care, Sighthill Campus, Edinburgh Napier University, 9 Sighthill Court, Edinburgh EH11 4BN, UK; l.anderson4@napier.ac.uk; 2 School of Computing, Merchiston Campus, Edinburgh Napier University, 10 Colinton Road, Edinburgh EH10 5DT, UK; h.koikkalainen@napier.ac.uk (H.K.); p.lapok@napier.ac.uk (P.L.); a.lawson@napier.ac.uk (A.L.); 3 Ageing and Health Research Group and Advanced Care Research Centre, Usher Institute, University of Edinburgh, Edinburgh EH16 4UX, UK; susan.shenkin@ed.ac.uk

**Keywords:** care home, foundation level barriers, digital solutions, digital health, connectivity

## Abstract

The care home sector has great potential to benefit from technological innovations and to be at the forefront of developing novel digital solutions to improve the experiences of care home residents, their families, and the staff caring for them. The COVID-19 pandemic exposed variability in digital capabilities and longstanding data challenges within the care home sector. Paradoxically, however, it also increased the use of digital tools and services to support residents and staff. There are, however, a number of barriers to sustained and widespread adoption of digital solutions by care homes. Here, the focus is on foundation-level barriers and the groundwork required to overcome them. Using data from three Scottish-based studies, foundation-level barriers to the adoption of digital tools and services faced by care homes are discussed. These main barriers are the need for robust basic internet connectivity; capabilities for digital data collection; access to data to inform and drive digital solutions; the need for trust in the use of resident data by commercial companies; and the danger that poorly coordinated strategies undermine efforts to build a care home data platform and the digital solutions it can support. Sustained and widespread adoption of digital solutions by care homes will require these foundation-level barriers to be addressed. Strong and stable data and digital foundations supported by sector-specific scaffolding are major prerequisites to the widespread adoption of digital solutions by care homes.

## 1. Introduction

Care home residents, their families, and the staff caring for them would benefit greatly from the widespread adoption of collaboratively developed digital health and social care technologies, such as telecare alarms and sensors, falls monitors, telehealth solutions, frontline point of care data capture, and online training and wellbeing support for staff. Better use of data and digital solutions will not only serve to enhance care services but can also derive economic benefits, reduce staff burden and enable better communication and closer working between health and care professionals [1,2,3]. Realising this potential is increasingly important as the ageing population and rising prevalence of chronic conditions will place a growing demand for care home services across the UK in the coming years. In August 2020, there were approximately 500,598 adult care home residents in the UK, of which 425,408 were in England, 14,935 in Northern Ireland, 35,989 in Scotland and 23,766 in Wales [4]. These figures are expected to rise significantly over the next two decades as it is estimated that the number of people aged 85 years and over will almost double by 2045 [5], and 40% of people in England and Wales will live and subsequently die in a care home [6]. Moreover, studies have shown that the level of disability and the complexity of health problems amongst care home residents is increasing, placing additional demands on care home staff and health professionals [7]. In contrast, the number of available hospital beds in the UK has decreased over the years [8,9] and is now only a third of the available care home spaces.

The development of digital tools for health and social care is advancing at a rapid pace [10]. These innovations range from simple tools, such as single-use applications or informational websites, to more complex solutions, such as mobile apps or devices integrated into formal healthcare systems or advanced data analytics tools for decision support [10]. However, while the availability of new digital technologies has grown rapidly in recent years, actual implementation in practice is still a recognised challenge within the social care sector [3,11]. Reported reasons for this include a lack of funding and resources, limited staff capacity and capability, a low level of knowledge and awareness among providers and care staff, ethical concerns, and resistance to change [1,3,12,13]. A recent review [14] found that, for half of the care providers, a lack of financial resources to invest in appropriate technology was a significant barrier to digital adoption, as well as a lack of time and resources to upskill staff, which was cited by a third of the providers. The review notes that the precarious financial environment in which the social care sector operates, characterised by short-term investments and spending review periods, impacts the sector’s ability to take a more strategic approach to longer-term digital investment and innovation. Some studies have emphasised the importance of the organisational context and culture of care homes for successfully implementing new initiatives and suggested frameworks for assessing a particular care home’s readiness for change [11,13,15,16]. While these studies have helped to understand some of the barriers to investment in and the implementation of new technologies in the social care sector, a deeper understanding of the foundational-level challenges is equally important for developing future innovations and enabling sustainable digital change.

Change, hastened and renewed by the pandemic, is what care homes will inevitably face—ready or not. In Scotland, for instance, current plans for a National Care Service will, once implemented, result in significant strategic and operational changes that will impact how care homes run their services [17,18]. The related construction of a National Digital Platform, which is a central record of health and care data for Scottish citizens, will enable the use of real-time data from health and care records [19,20]. Such changes will require substantive alterations and developments in the current technical architecture, integration capabilities and overall service infrastructure of health and social care. Designed to address the current technical landscape dominated by various individual IT systems and data silos, a national infrastructure based on open standards and with a centralised data platform at its core offers great potential for system interoperability and better use of health and social care data. As outlined by the Scottish Government strategy, it will provide opportunities for information capture and access at the point of contact; opportunities for research and innovation; more appropriate use of information—and any platform must be effective in the social care (including care homes), as well as healthcare environment.

To a large extent, this hoped-for data and digital transformation will stand, or fall, on the extent to which it is buttressed and supported by what happens ‘at ground level.’ Strong data and digital foundations are required to support the widespread adoption of digital solutions by care home providers and staff. By foundation level, we mean system level and structural aspects across the sector and within individual care homes. This commentary considers the question posed by this special issue—what are the barriers to the widespread adoption of digital solutions—and focuses on care homes and foundationlevel barriers. By outlining the foundation level barriers identified from three Scottish studies, the commentary provides insights relevant to care home managers, providers of electronic care planning software, local and regulatory authorities, and policymakers.

## 2. Research Studies

This commentary and discussion arises from insight gained from three studies undertaken by a multi-disciplinary research team based in Scotland with expertise in health and social care service evaluation, clinical research, data analytics, software systems development, and health informatics, including membership of the Care Home Innovation Partnership (CHIP) in Lothian, Scotland (LJ & SDS) [21]. While these studies have focused on the Scottish context, the issues identified are relevant to the adoption of digital solutions by care homes across the UK and other countries.

### 2.1. Care Home Data Platform

The Care Home Data Platform [22] study engaged six care homes within the Lothian region of Scotland to closely examine the data items collected and assessment tools in use for recording resident data. This work was carried out between July 2019 and January 2020, and the methods included reviewing data collection systems and documents, interviews with care home managers and discussions with key Scottish care home data stakeholders.

### 2.2. Landscape Assessment of Data and Digital Readiness of Scottish Care Homes (LADDeR)

The LADDeR study [23] centred around a survey of care homes for older people in the South East of Scotland (SE Scotland) to determine their current digital connectivity and data practises in care planning and medication management. The study took place from July 2021 to January 2022 and yielded frontline information from 110 care homes, representing 55% of SE Scotland’s homes and being representative of the geographic and sector spread.

### 2.3. Governance, Ethics, Access & Readiness through an Exemplar Demonstration (GEARED Up)

GEARED Up explored the milieu of data ethics and information governance that regulates the re-purposing of individual resident-level data, with a key focus on exploring current processes required by researchers and innovators to access such data to inform their work to improve and advance services. The methods included online research, discussions with topic experts, and completion of a data access application through the required processes. The study was carried out between January and June 2021. This work paralleled The Care Home Small Business Research Initiative (SBRI) Innovation Foundation Challenge [24], which was built on an earlier study [22] to assess options for establishing a Care Home Data Platform, as described above.

## 3. Foundation Level Barriers

### 3.1. Connectivity of Care Homes

Internet access is a basic, foundation-level technology infrastructure that all digital solutions will depend on to some extent. It is required not only for the use of essential online services, such as email and web-based applications, but also for accessing and maintaining services or applications that may also have some offline functionality. Software updates (including functionality upgrades, bug fixes, and security patches), secure multi-user access, data backups and recovery, and electronic data sharing with trusted external partners all rely on the ability to connect to the internet. Indeed, in the Digital Approaches in Care Homes Action Plan [25] (p. 7), the first feature of a digitally-enabled care home is ‘a superfast broadband connectivity, or the equivalent, into and within all areas of the care home’, highlighting the importance of this now almost omnipresent, yet still inconsistently functioning, technology for establishing strong digital foundations for care homes.

The LADDeR study found that whilst all care homes in SE Scotland were able to connect to the internet, the quality of the connection was less than optimal for most. 18% of the care homes reported poor connectivity and regular service interruptions, and a further 40% reported experiencing long loading times and occasional service interruptions. One in 4 (27%) reported that internet connection was only available in some parts of the care home. Half of the homes with only partial coverage were within the City of Edinburgh, indicating that an urban setting does not guarantee good connectivity.

### 3.2. Capabilities for Digital Data Collection

Despite the many recent advances in digitising the health and social care sector, as described, for example, in [10,26], many care homes are still at the bottom rung of the ladder when it comes to the use of digital data collection systems. Findings from the LADDeR study confirmed that paper-based systems predominate across all care homes of all sectors in SE Scotland. Only 35% of care homes in SE Scotland currently use an electronic care management system and two in five (43%) an electronic medication management software or system. Those with digital information systems were predominantly private sector homes. The cost of introducing digital systems was the most common reason given by responding care homes as to why they remain paper-based, and it not being the right time to invest in this was also reported.

There is also a lack of standardisation in the way data about residents and the care provided is specified and recorded. For example, individual data items often serve more than one purpose and can be required by different organisations, such as regulatory bodies and the Scottish Government, who specify similar data in different ways. The variety of data formats between homes is compounded by the lack of consistency in the assessment tools used to generate the data. The Care Home Data Platform study [22] found that whilst all care homes record similar information, such as mobility or risk of falls, the tools and assessments used to generate the data items are varied and, therefore, the data is not easily comparable.

Data linking and interoperability are also currently very limited, resulting in data silos and further reducing the potential for better data analytics [27]. It also leads to discrepancies in the quality of data available to care home staff, medical staff and researchers [28]. In addition, capturing the full complexity of care for individuals with multiple long-term conditions and high support needs will be essential for informing policy and practice but is at present made difficult due to the lack of systematic data collection in care homes [29].

### 3.3. Access to Data to Inform and Drive Digital Solutions

When considering the use of individual-level data for service development and technological innovation, it is essential that effective ethical and governance frameworks are in place. This will ensure that data-driven innovation in the sector is not hindered by a lack of clarity over data control and ownership, or by differing interpretations of the level of consent required from individual residents to allow their data to be used by commercial companies. Actual routes to governance and ethical approval of data to be used by those planning, designing or implementing new digital solutions for the care home sector are not clearly laid out. They are less well developed than those in place for NHS datasets, and, as a result, they can be excessively difficult to navigate.

Currently, there is no established system for the governance of care home data, which is held by a mixture of private companies, care regulators, and health and social care service providers [27]. Overall, the governance and ethics infrastructure around social care data is not nearly as well developed as that of NHS data sets. For example, there is no ethical or governance body that oversees all research in care home settings and accommodates the needs of public, voluntary and private sector providers, and no statement equivalent to website or paper notices in the NHS that data can be used anonymously for service development purposes (and that consent for this can be withdrawn).

Gaining access to social care data requires an understanding of several separate processes involving a number of different bodies that play a role in controlling data access, including the Integrated Research Application System (IRAS), NHS Scotland’s Public Benefit and Privacy Panel for Health and Social Care (NHSS HSC-PBPP), Health and Social Care Partnerships, and University ethics boards. Furthermore, data held within private-sector care homes often require data sharing agreements to enable access, which have to be established individually with each care home. This fragmented landscape of care home service provision and lack of a defined process for gaining permissions and access to data is a significant barrier to research, service evaluation and innovation work in the care home sector, and a more streamlined approach is needed to facilitate this work to bring service improvement.

### 3.4. The Need for Trust in the Use of Care Home Data

Ultimately, public and professional trust and confidence in the systems used to manage health and social care data will be critical to their rollout and success. This was demonstrated in 2021 with the attempted rollout of NHS Digital’s General Practice Data for Planning and Research (GPDPR) scheme in England, which raised concerns around the lack of transparency on data use [30], with the term ‘techlash’ having been coined to describe this issue [31]. Moreover, a recent report [32] found that service providers can often be uncertain about the advantages of data sharing, with some private sector providers being concerned about the commercial sensitivity of data that could expose or compromise their competitive advantage. However, the providers appeared to be generally more willing to share information when there were well-established and collaborative relationships in place with the local authorities. One route to establishing trust and confidence has been the creation of regional data safe havens within Scotland [33]. This centralised approach to managing, storing and handling access requests to healthcare data, led by trusted partners such as the NHS, academic institutions and government agencies, offers a practical solution from trusted partners that help to secure trust and confidence that data is handled respectfully, professionally, and securely. In addition, they enable the centralisation of data skills for linkage and analysis [2]. The SAIL (Secure Anonymised Information Linkage) Database in Wales is another example of this approach [34] that has yielded a rich source of data for researchers and policy makers. However, there are currently few trusted third-party intermediaries who could ingest data about individual care home residents and securely share it with appropriate and vetted data processors, reducing concerns about unethical uses [28].

### 3.5. The Need for Certainty and Coordination

**Figure 1:**
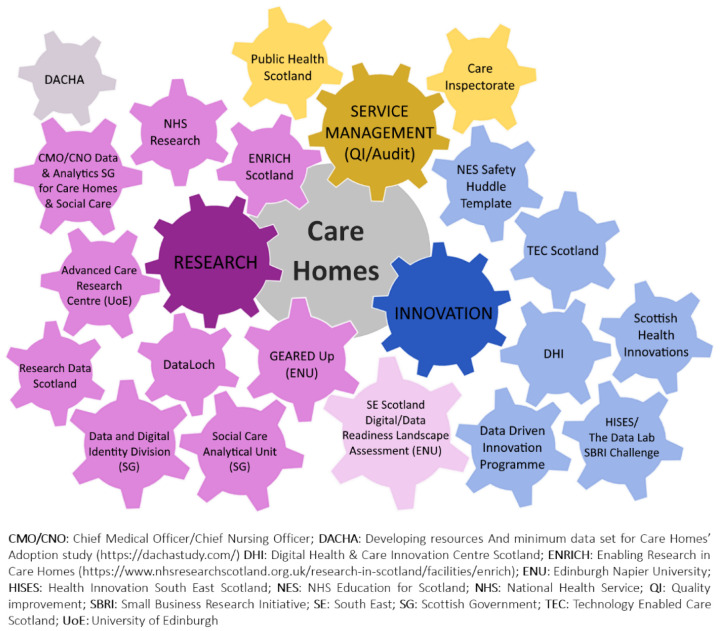
Multiple stakeholders are ‘gearing up’. A complex mix of stakeholders, in addition to providers, have an interest in care home data.

Multiple stakeholders in Scotland are now ‘gearing up’ with the aim to move forward and implement change to the digital landscape in health and social care. Figure 1 illustrates these stakeholders as multiple moving ‘cogs’ in the care home data wheel and illustrates that changes made by each of them will, in turn, impact each of the others. Unless coordinated tightly, changes implemented by one of the stakeholders may inadvertently ‘turn the cogs’ of the others with unintended consequences.

The shared aim of these stakeholders is to improve communications and efficiency of information handling and to enable wider uptake of digital solutions within the sector.

Many have been impelled by the impact of the COVID-19 pandemic on care homes and the data deficiencies that affected responses. Transformation of the collection, management and shared use of care home data, data-driven evidence, and digital innovation are core aspects of the COVID-19 recovery plans in Scotland [35] and also part of the continued development and adoption of digital solutions to key health and social care challenges as outlined by the Scottish Government in the last three years [19,20].

While this increased interest from a wide range of stakeholders will undoubtedly help drive change, there is a danger that a lack of coordination will result in duplicated efforts or, at worst, in incompatible ideas hindering each other’s progress. In Scotland, for example, 19 separate organisations and projects are now pursuing new ways to collect and use care home data either for research, innovation or service management purposes. These stakeholders represent a diverse mix of national policymakers, regulators, charitable organisations, academic researchers, clinicians and commercial innovators, each with different aims and plans for using care home data.

## 4. Conclusions

From three Scottish-based studies, five foundation-level barriers to the adoption of digital tools and services faced by care homes were identified. These main barriers are the need for robust basic internet connectivity; capabilities for digital data collection; access to data to inform and drive digital solutions; the need for trust in the use of resident data by commercial companies; and the danger that poorly coordinated strategies undermine efforts to build a care home data platform and the digital solutions it can support.

Despite the rapid development of data technologies and a renewed drive, post-COVID-19, for change and modernisation of health and social care data systems, significant foundation-level barriers still exist. If left unaddressed, they will continue to undermine the widespread adoption of digital solutions by care homes. Consistent and reliable internet connectivity is still largely lacking in Scottish care homes, and many homes continue to rely on paper-based systems for capturing and managing information about their residents. Without an equitable construction of these basic foundations, the digital transformation of the health and social care services in Scotland cannot be realised. Data connectivity and electronic data capture are fundamental to digital transformation, and this basic groundwork must be laid out first if the widespread adoption of digital solutions is to be supported.

The landscape within which this change is developing is busy and complex, with a mix of clinicians, social care providers and digital/technology innovators, all working within a framework of political, governmental, social, cultural and regulatory influence. Clear communication and coherence between local and national data initiatives, as well as wider whole-system coordination, from the foundations up, will be essential to ensure the identified wheel of stakeholders all turn in the same direction and at the same time.

As part of a renewed drive for change to modernise data collection systems within care homes and improve data quality, attention must also be given to the supporting infrastructures around data access and use, especially by commercial companies. Without realigning the access platform to become more streamlined, it is hard to envisage how the digital transformation of the sector will be realised.

Finally, the need for trust in these systems will be critical, and it is essential that this is inbuilt into the data platform. This will give care home staff trust in the systems they use; give patients and their families the confidence that their data is handled in the right way; to ensure data access requests are scrutinised appropriately, yet not to the extent that they introduce significant barriers; and to provide transparency in the uses of data and outcomes achieved. Public engagement, leadership, and ensuring that the processes, outcomes and advantages are communicated and understood better will be key to ensuring not only trust, but also the much-needed certainty and coordination in the digital transformation of health and social care services.

By outlining these foundation-level barriers to the use of digital systems in the care home sector, this commentary contributes to the topic of this special issue and provides important insights for care home managers, providers of electronic care planning software, local and regulatory authorities, and policymakers. Given the fast-paced changes in the technical architecture and infrastructure envisioned by the new strategies, there is not only an urgent need to consider the uneven and unstable data and digital foundations of care homes, but also the strength and solidity of the emerging structural supports to enable care homes to move up on the digital ladder.

## Data Availability

Not applicable.
